# Supplementary material for: Basal freeze-on generates complex ice-sheet stratigraphy
Source: Nat Commun. 2018 Nov 7;9:4669. doi: 10.1038/s41467-018-07083-3 (PMC6220257; doi:10.1038/s41467-018-07083-3)
Supplement: Supplementary file 1 — Supplementary Information [file 41467_2018_7083_MOESM1_ESM.pdf]

Supplementary Information
Basal freeze-on generates complex ice-sheet stratigraphy

by Leysinger Vieli et al.

Authors: G.J.-M.C. Leysinger Vieli*[1,3], C. Martín[2], R.C.A. Hindmarsh[2] and M. P. Lüthi[1]

[1] *University of Zurich, Department of Geography, Zurich, Switzerland*

[2] *British Antarctic Survey, Natural Environment Research Council, Cambridge, United Kingdom*

[3] *previously at Durham University, Department of Geography, Durham, United Kingdom*

Correspondence and requests for materials should be addressed to G.J.M.C.L.V. (email: gwendolyn.leysinger@geo.uzh.ch)

Supplementary Figures

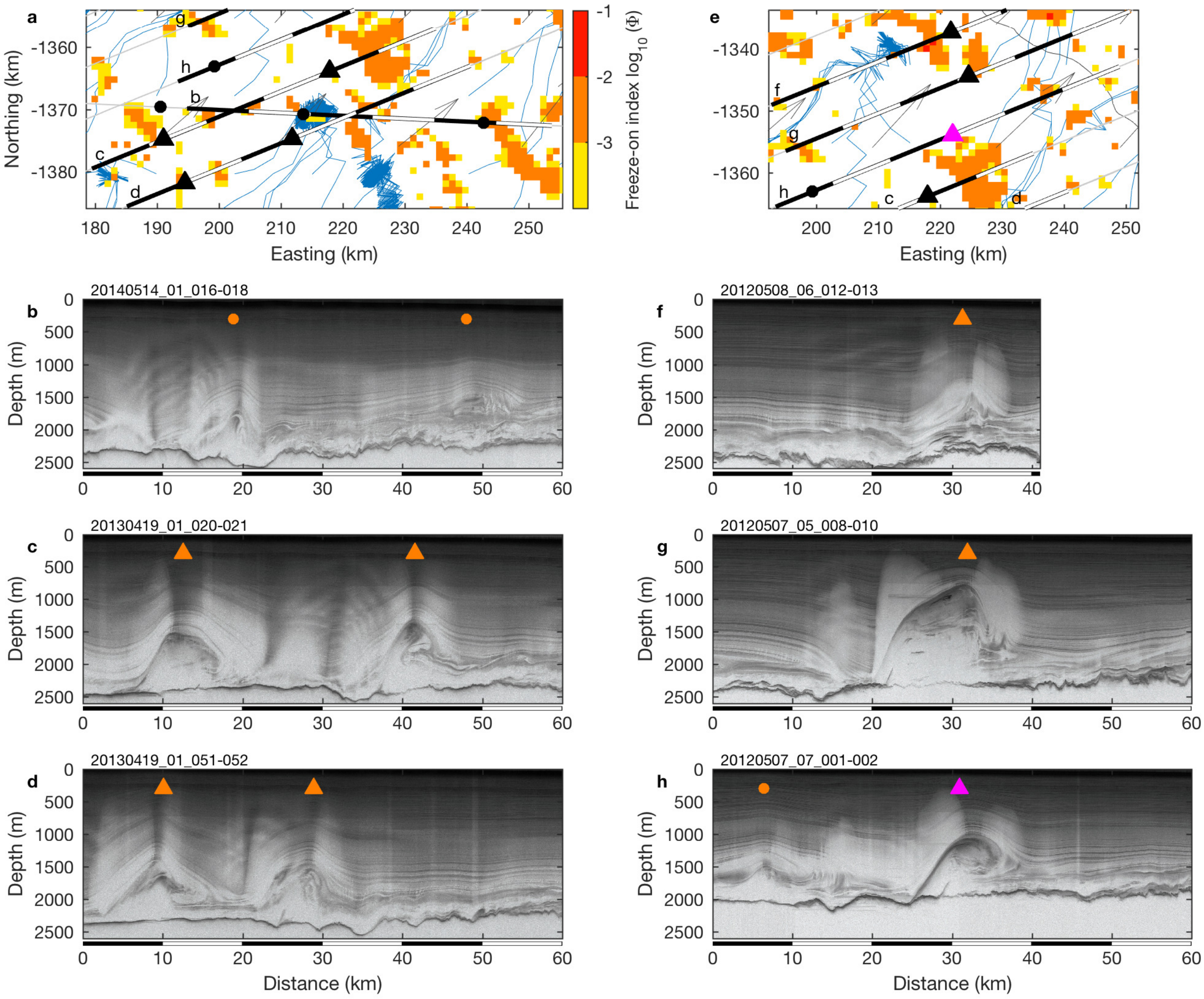

Supplementary Figure 1: **RES profiles of mapped plumes in North Greenland. a,e**, Enlargement of map area outlined in Supplementary Fig. 2 (magenta box), with **a** and **e** partly overlapping, showing freeze-on index Φ together with streamlines. Transects of RES profiles shown in **b-d, f-h** are highlighted with a 10 km scale bar. Plumes $\geq H/3$ (black-filled triangle) shown together with plumes $< H/3$ (black-filled dot). See Supplementary Fig. 2 for complete caption. **b-d, f-h**, Examples of RES profiles[1] $2010 - 2012$ (with corresponding data segment and frame number, and 10 km scale bar as displayed in **a,e**) showing internal layer-pattern and plume-like structures. Their apex (orange-filled dot or triangle) is chosen by taking the peak in internal layers of the meteoric ice above plume like features, which marks plume position on the maps in **a,e**, Figs. 1, 2 and Supplementary Figs. 2, 3. **f, g**, possibly showing effect of stacked plumes, where advected plumes that originate upstream are pushed up by local freeze-on, in combination with effects from three dimensional flow. **h**, Plume example (magenta-filled triangle) as chosen in Fig. 3a.

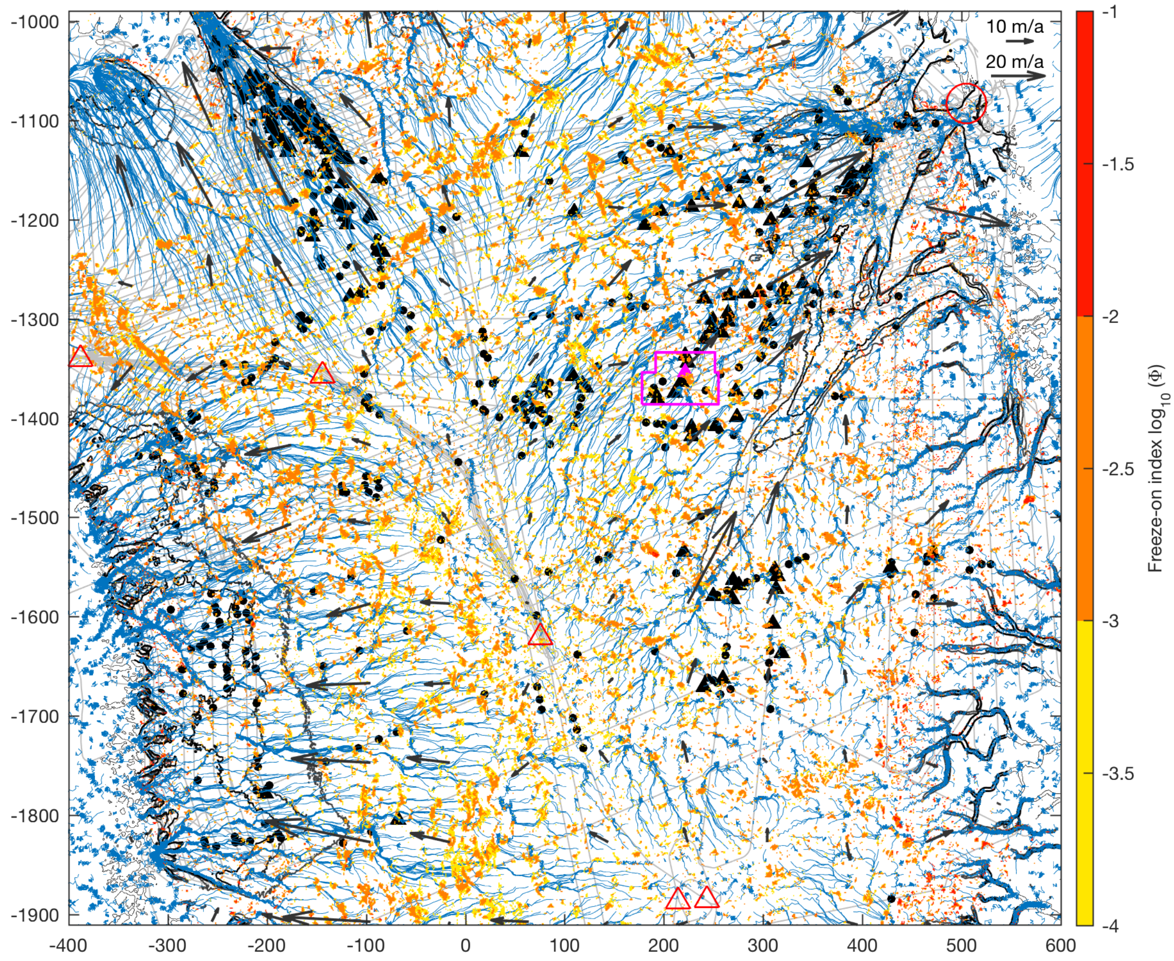

Supplementary Figure 2: **Plumes in relation to freeze-on index Φ and water paths.** Calculated freeze-on index Φ (yellow-orange-red; as in Fig. 2)) superimposed on the modelled basal-water flow path (blue; seeds on 12 km grid with 3 km step-size along streamline; see Methods section 'Estimate of water paths') along the hydrostatic-potential gradient. Map section and labeling as in Fig. 2. Direction and magnitude of ice-surface velocity[2] on 80 km grid (dark-grey arrows). Flow speed contours at 50, 75 and 100 m a^{-1} (dark-grey to black). Outlined area (magenta) shown as zoom in Supplementary Fig. 1.

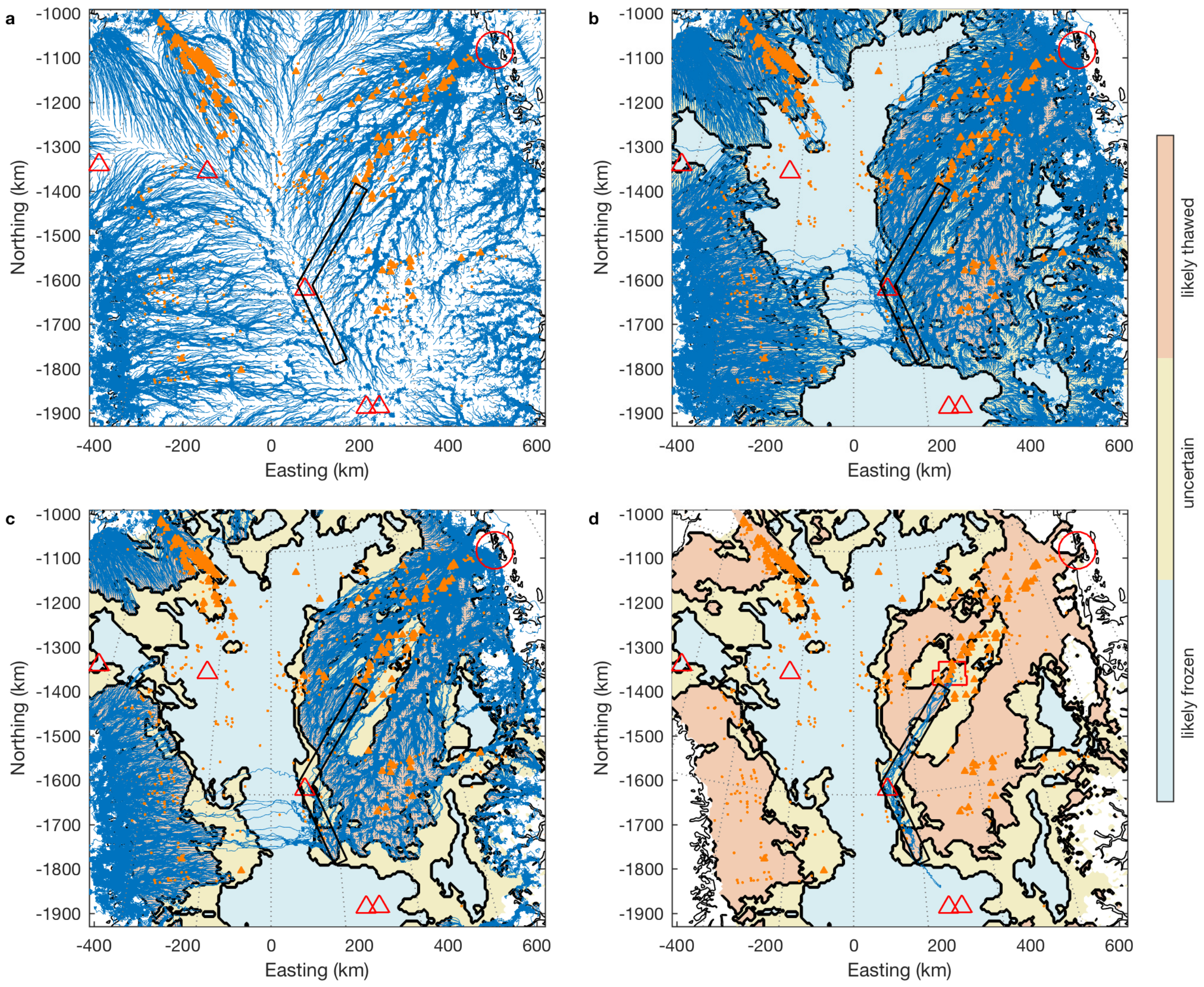

Supplementary Figure 3: **Modelled water paths in North Greenland.** Water paths (blue lines) calculated with *MATLAB streamlines* function[3] on a 1050 m topography grid, using a 5 km step-size along the streamline, shown together with plumes (orange-filled dots and triangles). **a**, Streamlines from seeds with 10 km spacing. **b**, Streamlines from seeds with 5 km spacing in areas of likely and uncertain thawed bed after MacGregor et al.[4], superimposed on thawed bed likelihood map. **c**, Streamlines from seeds with 5 km spacing in areas of likely thawed bed, superimposed on thawed bed likelihood map[4]. **d**, Streamlines (blue line) along the reversed hydraulic gradient using seeds at 1050 m spacing within a rectangular area (6 × 15 km) placed over the plume shown in Fig. 3a to obtain source region (black rectangular outline; see Method section 'Estimate of basal melt water'). See Fig. 1 for complete caption.

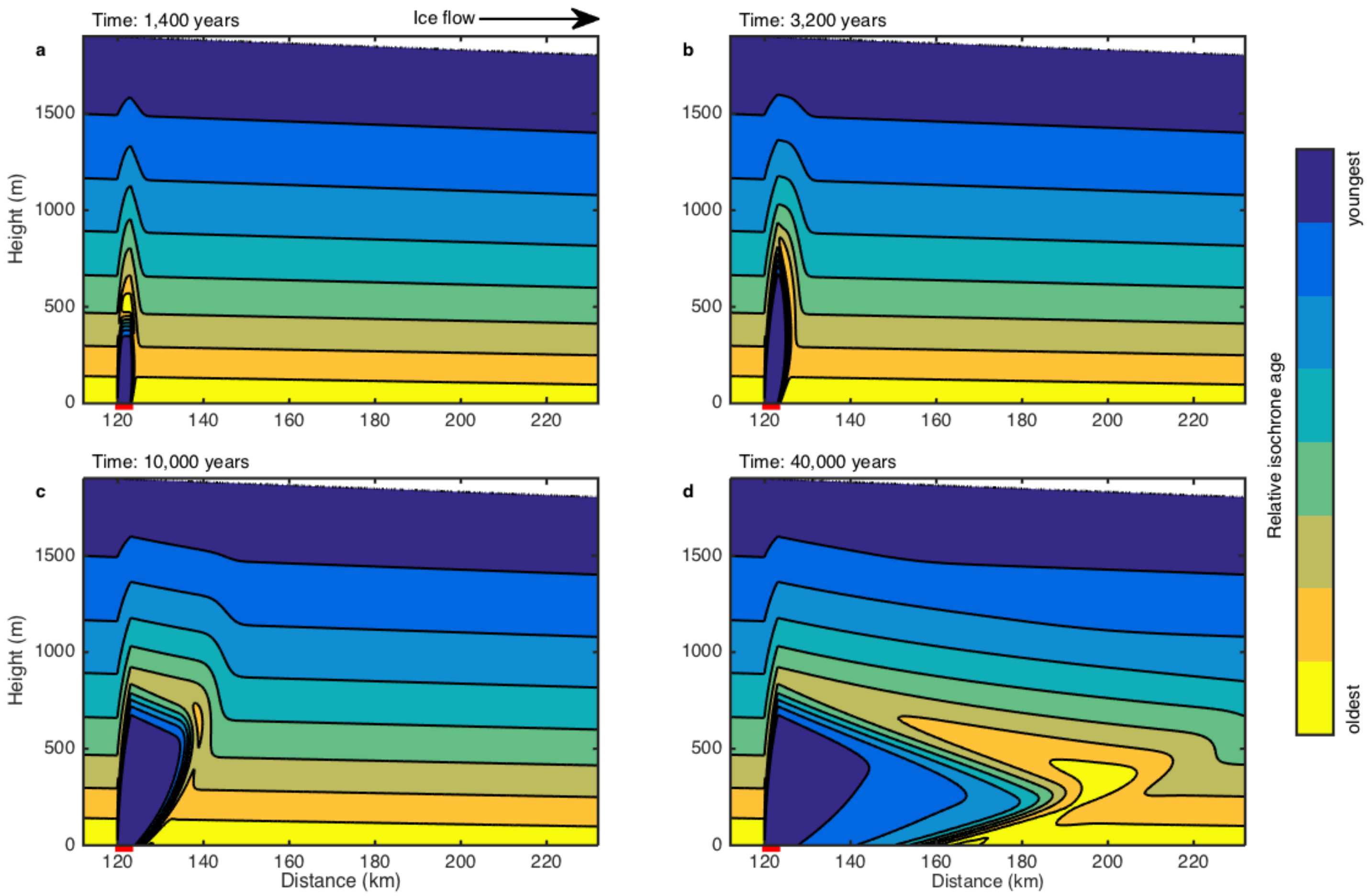

Supplementary Figure 4: **Modelled growth and evolution of a freeze-on plume. a-d**, Modelled contours of ice age (internal layers) using surface-accumulation rate $\dot{a} = 0.03$ m a^{-1} and slope $\alpha_s = 8.5 \times 10^{-4}$ (e.g. representative for Antarctica) with freeze-on over a 3 km accretion area with a rate of $\dot{f} = 0.3$ m a^{-1}. **a-b**, First stage of vertical growing after 1,400 and 3,200 years, respectively. **c-d**, Second stage of plume advection after 10,000 and 40,000 years, respectively, leading to internal layers being folded in front of the plume. Note this is an example with larger basal melt (0.01 m a^{-1}) outside the accretion area.

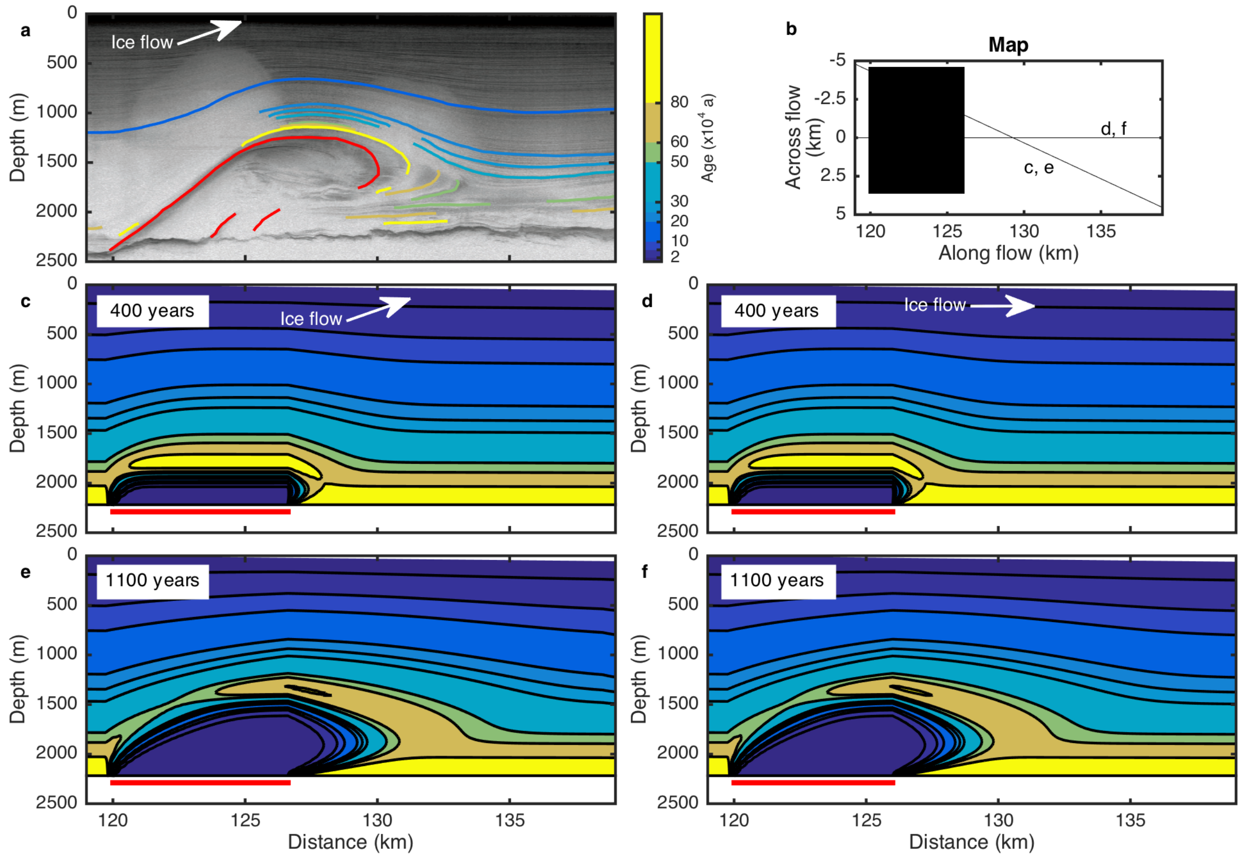

Supplementary Figure 5: **Observed and modelled internal plumes oblique to and along flow.** **a**, RES profile (20120507_07_001, 002)[1] along flight line (≈ 25° oblique to ice flow) with traced internal-layers as in Fig. 3b. **b**, Aerial view of model section with accretion area (black) and profile transects (black lines) oblique to (**c, e**) and along flow (**d, f**. **c and e**, Modelled internal layer-structure for a profile crossing accretion (red solid line) and plume-advection area (as in **b**) at an angle of 25°. **d and f**, Modelled layer structure for a profile crossing accretion (red solid line) and plume-advection area (as in **b**) along flow. Note, oblique to flow the plume shows greater elongation and therefore only 1100 years are needed to match the observed layer-architecture, but despite the angle still shows similar layer structure as along flow. See Fig. 3 for complete caption.

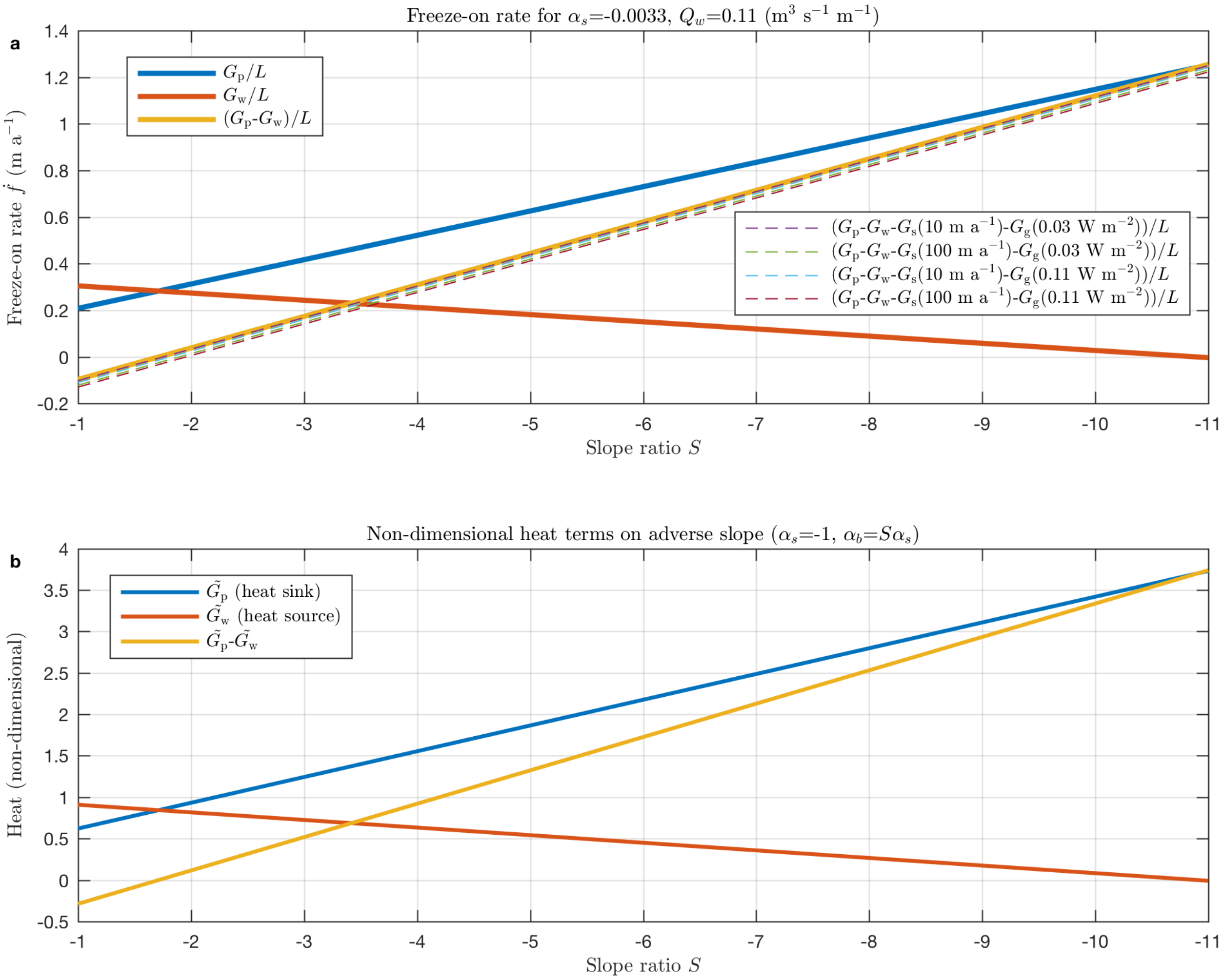

Supplementary Figure 6: **Relationship between heat terms and slope ratio.** Relationship of freeze-on between G_{p}, G_{w} and the difference $G_{\mathrm{p}} - G_{\mathrm{w}}$ for possible range of adverse slope ratios S (see equations (22) and (23)). **a**, Freeze-on rate $\dot{f}$ for heat terms depending on slope ratio when using the specific surface slope $\alpha_s = -0.003$ and water flux $Q_w = 0.11$ m^3 s^{-1} per unit width as used for the example in the text. In order to visualise the effect of the neglected heat terms on the freeze-on rate, all heat terms are included in the calculation using a lower and an upper value for both the heat from sliding G_{s} and from the geothermal heat flux G_{g}. For a velocity range of $10 - 100$m a^{-1} and using an ice thickness of $H = 2200$ m, G_{s}, is contributing $0.0019 - 0.0194$ m a^{-1} to freeze-on. Where a geothermeal heat flux range of $G_{\mathrm{g}} = 0.03 - 0.11$ W m^{-2} is contributing between $0.0031 - 0.113$ m a^{-1}. **b**, Relationship between the non-dimensional heat term components $\tilde{G}_{\mathrm{p}}$, $\tilde{G}_{\mathrm{w}}$ and the difference $\tilde{G}_{\mathrm{p}} - \tilde{G}_{\mathrm{w}}$ for possible range of adverse slope ratio S using $\alpha_s = -1$. Towards a stronger negative S (steeper α_b) the heat $\tilde{G}_{\mathrm{p}}$ required to maintain the water at the pressure-melting point increases while the heat $\tilde{G}_{\mathrm{w}}$ resulting from water flow declines towards zero. The extra heat $\tilde{G}_{\mathrm{p}} - \tilde{G}_{\mathrm{w}}$ needed to warm the water is obtained from latent heat released by the process of basal freeze-on.

Supplementary Tables

Supplementary Table 1: **Plume relationship with freeze-on index Φ.** The size of the (mapped) data sets are shown for both plume sizes as well as for the random data set. On a 1050 m grid for bed and surface topography, the position of streamlines starting from the plume area and calculated along the reversed ice-flow gradient have been compared with the location of freeze-on index Φ. Two sets for the maximum seed distance (area radius 1.5 and 3 km) and flow distance (5 and 10 km) from the plume have been used. The percentage shows the match between plumes and areas of freeze-on index for each data and experiment set. Note, the approximate error in the random set is $\pm 1\%$.

	Number of plumes		
	159	443	10,000
Radius and upstream distance	$\geq H/3$	$< H/3$	random
1.5 km and 5 km	75%	70%	61%
1.5 km and 10 km	88%	80%	76%
3 km and 5 km	87%	87%	80%
3 km and 10 km	96%	93%	89%

Supplementary Table 2: **Relative plume mismatch with freeze-on index Φ between observed and random data set.** The data set and results are taken from Supplementary Table 1 to calculate the difference in percentage for plumes with no freeze-on area relative to the expected value in mismatch obtained from the random set of 10,000 plumes. Note, the approximate error in the random set is $\pm 1\%$.

	Compared to random set			
	Difference in mismatch		Relative difference	
Radius and upstream distance	$\geq H/3$	$< H/3$	$\geq H/3$	$< H/3$
1.5 km and 5 km	14%	9%	36%	23%
1.5 km and 10 km	12%	4%	50%	17%
3 km and 5 km	7%	7%	35%	35%
3 km and 10 km	7%	4%	64%	36%

Supplementary References

[1] Leuschen, C. *et al.* IceBridge MCoRDS L1B geolocated radar echo strength profiles, version 2. Boulder, Colorado USA: National Snow and Ice Data Center. http://dx.doi.org/10.5067/90S1XZRBAX5N (2014, updated 2016).

[2] Joughin, I., Smith, B., Howat, I., Scambos, T. & Moon, T. Greenland flow variability from ice-sheet-wide velocity mapping. *J. Glaciol.* **56**, 415–430 (2010).

[3] The MathWorks, I. *MATLAB*. Natick, Massachusetts, United States, release 2014b edn. (2014).

[4] MacGregor, J. A. *et al.* A synthesis of the basal thermal state of the Greenland Ice Sheet. *J. Geophys. Res. Earth Surf.* **121**, 1328–1350 (2016).
