# Supplementary material for: Basal freeze-on generates complex ice-sheet stratigraphy
Source: Nat Commun. 2018 Nov 7;9:4669. doi: 10.1038/s41467-018-07083-3 (PMC6220257; doi:10.1038/s41467-018-07083-3)
Supplement: Supplementary file 3 — Description of Additional Supplementary Files [file 41467_2018_7083_MOESM3_ESM.pdf]

Description of Additional Supplementary Files

Supplementary Movie 1

Description: Animation of growth and evolution of freeze-on plume. Along flow modelling of freeze-on as in Fig.3c,f,i. Modelled internal layer-structure purely by internal deformation for zero to 4900 years after switch on of freeze-on, with freeze-on $\dot{f} = 0.8\ \mathrm{m\ a^{-1}}$over $6\ \mathrm{km}$ accretion area (starting at $120\ \mathrm{km}$), for a surface-accumulation rate $\dot{a} = 0.1\ \mathrm{m\ a^{-1}}$, zero accretion and a surface slope $\alpha_{\mathrm{s}} = 0.003$. Age contours (at $0,\ 2,\ 6,\ 10,\ 20,\ 25,\ 30,$ ... 80×10^4 years; blue to yellow).
